# Applying human-centered design to identify needs and generate recommendations to inform an integrated maternal health demonstration model

**DOI:** 10.3389/fpubh.2026.1936761

**Published:** 2026-09-23

**Authors:** Amanda Castelo Saragosa, Alyson Schenck, Kaelia Lynn Saniatan, Nicole Taylor, Hailey Hernandez, Keiasha Carlton, Tracy Carver, Nadia Gomez, Gabriela Buccini

**Affiliations:** 1 School of Public Health, University of Nevada, Las Vegas, NV, United States; 2 Comagine Health, Seattle, WA, United States; 3 University of Nevada Las Vegas Kirk Kerkorian School of Medicine, Las Vegas, NV, United States

**Keywords:** community engagement, human-centered design, maternal health, maternal health services, qualitative research

## Abstract

**Background:**

Maternal mortality continues to rise in concerning rates in the United States. Maternal mortality risks are increased by clinical factors like cardiovascular disease or infection and nonclinical factors like food insecurity, unequal access to healthcare, and systemic racism. Integrated care coordination programs aimed at addressing care needs are emerging as best practices to improve maternal health outcomes at and post birth. Our goal was to (i) evaluate the care needs during pregnancy and postpartum and (ii) formulate recommendations for integrating a clinic-level model and community-level model to develop an integrated maternal health services model.

**Methods:**

Human Centered Design (HCD) activities were used to identify the care needs from pregnancy through postpartum involving 35 participants across three groups. Group 1 included community members; Group 2, clinicians and healthcare providers from an academic obstetric clinic; and Group 3, care coordination and social service providers. Each group completed three to four HCD sessions between April to July 2024. An inductive qualitative analysis of HCD outputs captured frequency, complementary, expansion, and divergence among groups, which subsequently informed the recommendations for designing the model.

**Results:**

Four categories captured the recommendations for designing the IMHS program: (1) Pregnancy needs (e.g., center the care of the pregnant women and their social support), (2) Pregnancy specialist needs (e.g., include doulas, midwives, and other specialties within care coordination), (3) Community system needs (e.g., create coordinated outreach systems to reach populations), and (4) Healthcare system needs (e.g., provide skill-based training for clinical staff).

**Conclusion:**

Recommendations for addressing pregnancy, specialist, community, and healthcare needs should be integrated into the design of the IMHS demonstration program to improve the care of pregnant women in Southern Nevada.

## 1 Background

Maternal mortality, defined as the death of a woman during pregnancy or within 42 days after the end of pregnancy, due to pregnancy-related complications or the mismanagement of pregnancy (1), continues to rise in the United States (2). In 2024, the maternal mortality rate in the United States was 17.9 deaths per 100,000 live births (3), which is more than double the rate of most other high-income countries like Germany, Japan, and Sweden (4). Maternal mortality disproportionately affects Black women, who have higher maternal mortality rates (49.5 per 100,000 live births) compared to White women (19.0 deaths per 100,000 live births) (4, 5). Clinical factors such as cardiovascular disease, hemorrhage, and infection are leading causes of maternal mortality (5, 6). Likewise, social determinants of health, which refers to nonclinical conditions, such as food or housing insecurity, lack of transportation, systemic racism, unequal access to healthcare, and disparities in care quality can significantly exacerbate the risks of maternal mortality (7, 8).

The American College of Obstetricians and Gynecologists (ACOG) recommends a comprehensive risk assessment during prenatal and postpartum care that should assess both clinical and nonclinical factors to reduce pregnancy-related deaths and ultimately maternal mortality (53). Implementing a comprehensive patient-centered risk assessment requires integrated care coordination at both clinical and community levels to establish a safety net that addresses both clinical and nonclinical maternal risk factors (9, 53). For example, the Maternal Medical Home Model, a clinical-level model that provides referrals for maternal clinical needs throughout pregnancy and postpartum proved to be effective in New York, Colorado, North Caroline, Colorado (10–12). On the other hand, the Pathways Community HUB, a community-level model that assists individuals in need by identifying their risk factors and connecting them to necessary clinical, social, and behavioral services was effective in reducing preterm birth (13). Combining these two evidence-based models into an integrated program aimed at addressing maternal clinical and social needs may be an emerging best practice to improve maternal health outcomes both during and after pregnancy. However, to date, there is a lack of recommendations on how to design an integrated maternal health program that involves diverse voices, such as clinicians, community services, and individuals with lived experience.

In 2023, the Health Resources and Services Administration (HRSA) funded the Integrated Maternal Health Services to develop integrated demonstration models aimed at improving maternal health (52). Clark County is the most populous county in Nevada, with a maternal mortality rate of 30.2 deaths per 100,000 live births (14), which is significantly higher than the national average and places the state among those with the highest rates in the United States. Because of this, Clark County, Nevada was selected as one of the locations to pilot the Integrated Maternal Health Services demonstration project. This manuscript aimed to describe the participatory process used to (i) evaluate care needs during pregnancy and postpartum and (ii) formulate recommendations for integrating the Maternal Medical Home and Pathways Community HUB models in Clark County, Nevada.

## 2 Methods

### 2.1 Study design

This is an exploratory, qualitative study that utilized human-centered design (HCD) framework to engage participants in identifying pregnancy and postpartum care needs of women in Clark County, Nevada. HCD is a structured framework that places individuals at the center of the problem-solving process, engaging them in developing solutions that address their identified needs (15–18). HCD enables studies to transition from research to application, yielding strategies that are genuinely beneficial to the intended recipients and can maximize their impact in the community served (17, 19, 20). In this study, HCD was operationalized using the LUMA system of innovation, which organizes HCD methods into three core design skills: Looking, Understanding, and Making (21). These skills support understanding participants and their experiences (looking), identifying patterns and framing problems (understanding), and generating potential solutions (making) (21). The LUMA system guided the selection and sequencing of HCD activities used with the participants in this study. This study was reported using Consolidated Criteria for Reporting Qualitative Research (COREQ) as shown in Appendix A (22).

### 2.2 Study setting

This study was conducted in Clark County, Nevada, which is home to 2.3 million inhabitants, accounting for 73% of Nevada’s total population (23). Approximately 9.4% of women in Nevada reported late/no prenatal care, which is much higher than the state (8.1%) and the nation (7%) (23). In 2024, it was found that the rate of pregnancy-associated deaths in Nevada was 98.4 deaths per 100,000 live births, with 76% of the deaths occurring in Clark County (DHHS, 2024). Additionally, the maternal mortality rate in Clark County is 30.2deaths per 100,000 live births, which is higher than the state rate (25.7) and the national rate (17.9) (3, 14). One of the significant contributing factors to poor maternal health outcomes includes health care access barriers and workforce shortages (24). Due to these higher rates and care barriers, Clark County was selected as one location to develop and pilot the integrated care coordination demonstration project.

### 2.3 Research team

HCD sessions were facilitated by six co-authors (ACS, AS, HH, NT, KC, NG) who obtained HCD Practitioner certification from LUMA Institute. All researchers involved in the analysis (ACS, AS, NG, TC, GB) had prior experience in research and qualitative analysis, as well as experience working in community-based research and evaluation projects. Authors are affiliated with UNLV School of Public Health (ACS, KS, GB), UNLV Kirk Kerkorian School of Medicine (KC, NG), and Comagine Health (AS, TC, NT, HH). Authors GB and NG have extensive experience working in maternal health research and pregnancy care in Clark County, respectively, and their interpretations of the findings are informed by this context.

### 2.4 Participant selection

Three groups of participants were recruited to participate in the HCD activities. All participants across the three groups currently or previously resided, practiced, and/or served within Clark County. Sampling techniques varied based on participant type.

#### 2.4.1 Group 1: community member group

The first group consisted of community members; the inclusion criteria for this group were individuals who were doulas, community health workers, pregnant, or new mothers. Purposive sampling was used to recruit doulas and community health workers based on their knowledge of Clark County’s perinatal resource landscape, and many have worked with the authors and research team in previous projects. Pregnant women or new mothers were recruited through volunteer sampling via flyers and were provided a stipend of $100 for each activity they participated in.

#### 2.4.2 Group 2: maternal medical home group

The second group consisted of physicians, providers, consultants, or program evaluators. Inclusion criteria for this group included any staff member or anyone who works with the participating Obstetric and Gynecology clinic who have knowledge on Clark County’s maternal healthcare landscape. Purposive sampling was used to recruit clinic staff members based on their relevant knowledge and experience. Snowball sampling was then used, where recruited clinic staff referred outside physicians, providers, consultants, and program evaluators who work with pregnant women or mothers.

#### 2.4.3 Group 3: southern Nevada pathways HUB group

The third group consisted of staff members or organizations that work with the Southern Nevada Pathways HUB, which is a Pathways Community HUB model that was developed specifically for Southern Nevada. The inclusion criteria for this group was any staff member of the Southern Nevada Pathways HUB (e.g., care coordinators) and community partners who work directly with them (e.g., birth support, social services, community organizations). Purposive sampling was used to recruit participants based on their knowledge of the Southern Nevada Pathways HUB and Clark County’s resource landscape.

### 2.5 Data collection

To identify pregnancy and postpartum needs, qualitative data was collected from each group based on the HCD activities prepared for them. HCD activities were selected from the LUMA system based on the objectives of each participant group and the type of information the research team sought to obtain. Consistent with LUMA’s three design skills, activities were selected to first identify participants’ experiences and perspectives (Looking), then synthesize and prioritize identified needs and barriers (Understanding), and lastly generate potential strategies and solutions for integrating maternal care and community-based services (Making). Activities were adapted and sequenced to facilitate exploration of pregnancy and postpartum experiences, identification and prioritization of care needs and barriers, and development of potential solutions. A full description of HCD sessions for each group, including HCD activities conducted, objectives from the LUMA institute, HCD activity goals, methods, and outputs can be found in Tables 1–3. Data was collected between April 2024 and July 2024 through in-person meetings or virtual meetings via Zoom. Each meeting lasted 1.5–2 h long.

**Table 1:** Description of human-centered design (HCD) sessions with group 1- community members, 2024.

HCD session	Activity	Activity objective (LUMA)	HCD activity goal	Methods	Output	Data analysis	Finding(s)
Session 1	Persona Profiles	They can help your team remember and discuss the people you are serving. As a reference for generating ideas, prioritizing features, or discussing trade-off decisions, personas are a valuable tool for decision making.	To create personas that assist the team in centering the women served by the model, and to gain an understanding of these individuals’ needs, experiences, behaviors, and goals.	Participants drafted profiles of fictional characters, relying on their own firsthand pregnancy experiences as well as their experience supporting pregnant women to compile these descriptions.	Physical: Individual persona profiles created by participants.	Qualitative thematic analysis of the persona profiles. Identified themes that emerged from the different profiles.	“Themes” of the struggles that individuals face. The themes with the most items included in the personas were: Goals for delivery (*n* = 5), Availability of resources (*n* = 4), experiencing misinterpretation (*n* = 4). Representative Quote(s): Goals for delivery: “Have a safe and healthy delivery” Availability of resources: “I felt very lost on my journey to getting pregnant and there was an extreme lack of resources available to me” Experiencing misinterpretation: “I was accused of causing my preterm labor, which was untrue”
Session 2	Experience Diagramming	Working as a group, map the experience of the people your company serves, including people, places, systems, and information they use to complete tasks.	To illustrate the “journey” of a pregnant woman and mothers within the healthcare system and identify the positive and negative experiences they have, including identifying where in the journey there are opportunities for improvement.	Participants laid out their pregnancy and postpartum experiences chronologically along a timeline, with the x-axis of the diagram indicating the emotional content of the experience by its position on the page vertically, with positive emotional experiences placed towards the top of the page and negative emotional experiences along the bottom of the page.	Virtual: Experience diagram that maps out the journey of pregnant women and mothers with votes of the top two experiences from participants.	Qualitative thematic analysis of the experience diagram. Grouped the different experiences by themes (i.e., coordination of care, interaction with staff, etc) and the top 2 experiences that the participants wanted to focus on.	Themes of individuals’ experiences. Representative Quote(s): The key negative experiences participants voted to move forward: “No preparation for mom after birth” and “Lack of resources.”
	Visualize the Vote	A quick poll of collaborators to reveal preferences and opinions	To illuminate the top two points of a pregnant women journey that the group wants to further focus on	Participants used sticky notes to pick their top 2.			
	Statement Starters	As their main objective, Statement Starters encourage restating problems as invitations for exploration. Instead of being dictatorial, leaving little room for deviation from a predetermined type of solution, these phrases ask your team to consider various alternatives.	To identify and frame the problem statements that community members advise the research team to prioritize in designing the model.	Using 2 negative experiences identified from the experience diagram, participants engaged in brainstorming multiple ways to frame these experiences as problem statements, and landed on the statements they felt best framed the problems they recommended the model prioritize.	Virtual: A list of statement starters for two experiences identified in the previous activity with votes from the participants.	Qualitative thematic analysis of the statements (not picked by group) and the top 3 problem framing statements.	Themes of statement starters. Representative Quote(s): The three priority statements chosen were: “How might we create cultural competency/humility within hospitals/ clinics,” “How might we educate mothers on how to take care of themselves after birth?” and “How might we educate mothers on their bodies?”
	Visualize the Vote	A quick poll of collaborators to reveal preferences and opinions.	To illuminate the top three statement starters that the group believed the design group should focus on.	Participants used sticky notes to pick their top 2.			

This table includes a description of the activities, activity goals, methods, outputs, analysis, and findings for each session done with Group 1.

**Table 2:** Description of human-centered design (HCD) sessions with group 2- maternal medical home, 2024.

HCD session	Activity	Activity objective (LUMA)	HCD activity goal	Methods	Outputs	Data analysis	Findings(s)
Session 1	VIP mapping	A way of diagramming the network of people who have a vested interest in a given system	To consider the landscape of pregnancy and postpartum care, identifying individuals impacted by the model and the interconnections between these individuals.	Participants considered the landscape of pregnancy and postpartum care and mapped out all of the individuals that a pregnant woman and mothers interacted with during their care. Participants then drew connections between individuals listed, labeling the interconnections between these individuals.	Physical: A network diagram of the individuals involved in pregnancy and postpartum care with labels showing the relationships between them.	Qualitative thematic analysis on the network diagram. Combined the two maps and combined similar stakeholders. Placed these stakeholders under different groups that they represent.	*n* = 9 different groups of stakeholders placed onto a diagram that shows the largest and smallest groups: Social support, built environment, social services, pregnancy specialist, other specialists, mental health, physicians, social needs, clinic staff.
	What’s on your radar	An exercise in which people plot items according to personal significance.	To identify the priorities of participants in relation to the care of pregnant women and mothers.	Participants listed their top 3 priorities for their pregnant patients and mothers by placing these priorities onto a radar diagram with primary priorities being placed in the center, and secondary and then tertiary priority stickies placed on the corresponding outer circles. Participants shared details around each priority as they placed it on the radar diagram.	Physical: A Radar diagram with each slice representing an individual and the sections showing the primary, secondary, and tertiary priorities of that individual.	Qualitative thematic analysis on the radar diagram. Combined like priorities. Organized them onto a socioecological diagram that shows the primary, secondary, and tertiary priorities.	Diagram depicting their primary, secondary, and tertiary priorities. The most common primary concern was access to care (*n* = 4). The most common secondary concerns were learning and education (*n* = 2) and emotional support during/after delivery (*n* = 2). Representative Quote(s): Primary: “Access to care. Pregnant, now what?” Secondary: “Learning (the healthcare system and available services)” and “Support/emotional support (during/after)”
Session 2	Experience diagramming	Working as a group, map the experience of the people your company serves, including people, places, systems, and information they use to complete tasks.	To illustrate the “journey” of a pregnant woman and mothers within the healthcare system and identify the positive and negative experiences from the perspective of the participants, including identifying where in the journey there are opportunities for improvement.	Participants laid out pregnancy and postpartum care experiences chronologically along the x-axis of a pregnancy timeline, indicating the emotional content of the patient experience by its position on the page vertically, with positive emotional experiences placed towards the top of the page and negative emotional experiences along the bottom of the page.	Physical: A drawing of an experience diagram that depicts the journey of pregnancy and postpartum care with sticky notes indicating the areas that the group wants to target for solutions.	Qualitative thematic analysis on the experience diagram. Grouped the different experiences by themes (i.e., coordination of care, interaction with staff, etc) and the top 2 experiences that the participants wanted to focus on.	Themes of individuals’ experiences. Representative Quote(s): The priorities that emerged from discussion were: “Health education for patients and families” and “Improving access to care and resources to decrease financial burden.”
	Visualize the vote	A quick poll of collaborators to reveal preferences and opinions.	To illuminate the top two points of a pregnancy journey that clinicians would like to target for problem-solving.	Participants used sticky notes to pick their top 2.			
Session 3	Abstraction laddering	A way of reconsidering a problem statement by broadening or narrowing its focus.	To identify and frame 2 problem statements that the group would like to use as starting points for crafting solutions in the design phase of the project.	Participants chose an initial problem statement and reworked that statement to determine the best framing and scope of the problem. Participants first moved up a ladder diagram and considered the problem in more abstract ways by asking “why” they wanted to address this problem. Participants then moved down the ladder asking “how” and considering the problem in more concrete ways.	Physical: Two drawings of ladders, each with an original statement of the target area in the center, and reframing going up (why) and down (how) the ladders. Each ladder had sticky notes indicating which problem frame the group wanted to move forward with creating solutions for.	Qualitative thematic analysis of the different problems (not chosen by the group)	“Themes” of the problems identified. Representative Quote(s): “How might we perform risk assessments for underserved populations?” “How might we make access to care available to the most vulnerable populations?”
	Visualize the vote	A quick poll of collaborators to reveal preferences and opinions.	To illuminate the top two problem statements that the group would like to brainstorm solutions for.	Participants used sticky notes to pick their top 2.			

This table includes a description of the activities, activity goals, methods, outputs, analysis, and findings for each session done with Group 2.

**Table 3:** Description of human-centered design (HCD) sessions with group 3- Southern Nevada Pathways HUB, 2024.

HCD session	Activity	Activity objective (LUMA)	HCD Activity Goal	Methods	Outputs	Data analysis	Findings(s)
Session 1	Problem tree analysis	Using the tree as a metaphor, you separate the causes (roots) from the effects (branches) of a central issue (trunk). It provides a structured way for your team to reveal concerns, discern causes from symptoms, and potentially frame problem statements in a new and better way.	To generate an understanding of pain points and barriers to care that pregnant women and mothers face, and the impact of those barriers.	Participants considered challenges connecting pregnant women and mothers to needed services. Participants placed sticky notes listing root causes along the tree roots, and sticky notes listing the impacts of this problem on the branches. Participants subsequently discussed the items they included and voted on the top 2 most impactful root causes that they wanted to move forward with to create problem framing statements.	Virtual: Problem tree with root causes and impacts (effects) and votes from participants.	Qualitative thematic analysis of the problem tree problems (not picked by group) and the top 2 causes/impacts.	Themes of root causes and impacts. Representative Quote(s): The barriers that the group voted to move forward with were “limited resources,” “lack of support system,” and “not understanding what programs and services exist.”
	Visualize the vote	A quick poll of collaborators to reveal preferences and opinions.	Participants used dots on MURAL to pick their top 2.	The top two root causes that the group found most influential and would like to move forward with.			
Session 2	Statement starters	As their main objective, Statement Starters encourage restating problems as invitations for exploration. Instead of being dictatorial, leaving little room for deviation from a predetermined type of solution, these phrases ask your team to consider various alternatives.	To identify and frame the problem statements that address the priority root causes identified.	Participants identified the top root causes from the Problem Tree and framed those barriers as problem statements. Participants then engaged in brainstorming multiple ways to frame those problem statements, and landed on the statements they felt best framed the problems to move forward into solutioning.	Virtual: A list of statement starters for two root causes with votes from the participants.	Qualitative thematic analysis of the statement starters (not picked by group) and the top 4 problem framing statements.	Themes of statement starters. Representative Quote(s): The statements participants chose to move forward with were “How might we engage the whole family in prenatal care?” “How might we create peer support within clinics?,” “How might we teach more self-reliance, confidence, and self-efficacy?” and “How might we use CHWs to increase support?”
	Visualize the vote	A quick poll of collaborators to reveal preferences and opinions.	Participants used dots on MURAL to pick their top 2.	The top two problem framing that the group liked best and would like to move forward with.			
	Creative matrix	If you want to generate many wide-ranging ideas in a short amount of time, the structure of a Creative Matrix is useful because it stimulates cross-pollination by providing a framework for generating new ideas where topics intersect.	To brainstorm potential solutions to the top 2 problem framing statements identified in session 2	Participants were presented with a matrix with relevant categories along the left axis and problem statements on the top axis. Participants brainstormed solutions by placing ideas in each square that address the problem at the top and correspond to the category along the left. Participants then voted on top 4 solutions they would like to consider incorporating into the design phase.	Virtual: Creative matrix with potential solutions with votes from the participants.	Qualitative thematic analysis of the creative matrix. Specifically of the solutions (not picked by group) and the top 2 solutions.	Themes of different solutions. Representative Quote(s): The solutions participants voted to prioritize were “Recruit and train folks from the community you are trying to serve” and “Offer training/classes/support in multiple ways in person/virtual, one on one, group, etc.”
	Visualize the vote	A quick poll of collaborators to reveal preferences and opinions.	Participants used dots on MURAL to pick their top 2.	The top 3 solutions the group liked best and believe the workgroup should focus on.			

This table includes a description of the activities, activity goals, methods, outputs, analysis, and findings for each session done with Group 3.

#### 2.5.1 Group 1 data collection

Data collected from Group 1 were three outputs from five HCD activities (Table 1). The first output was physical persona profiles detailing some pregnancy and postpartum experiences that participants completed with pen and paper. The second output was an experience diagram that maps out the experience of pregnant women and mothers that was collected virtually through the web-based collaboration platform Mural. The third output was a list of statement starters on negative pregnancy or postpartum experiences that was collected virtually through Mural.

#### 2.5.2 Group 2 data collection

Data collected from Group 2 were four outputs from six HCD activities (Table 2). All outputs were collected physically, where participants developed them using markers and large paper canvases. The first output was a physical pregnancy and postpartum network diagram. The second output was a physical radar diagram of pregnancy and postpartum priorities. The third output was a physical experience diagram that maps out the experience of pregnant women and mothers. The fourth output was a drawing of abstraction ladders that framed the priority pregnancy or postpartum problems that the group wanted to focus on.

#### 2.5.3 Group 3 data collection

Data collected from Group 3 were three outputs from six HCD activities (Table 3). All outputs were collected virtually through Mural. The first output was a drawing of a problem tree that illustrates the root causes and impacts of barriers to pregnancy and postpartum care. The second output was a list of statement starters on negative pregnancy or postpartum experiences. The third output was a creative matrix that includes potential strategies for addressing pregnancy and postpartum experiences.

### 2.6 Data management

Physical and virtual data were collected, none of which had personal or identifiable data. Physical data was collected via pen and large paper canvases and was immediately developed into virtual documents and spreadsheets by the research team and stored in a secure folder in Google Drive. The physical copies were disposed of after being made into virtual files. Virtual data was collected through Mural, and transferred to Google Drive by the research team. Data from each group were stored in separate folders in Google Drive.

### 2.7 Data analysis

First, descriptive statistics (e.g., frequencies and percentages) were calculated to summarize participant roles within each group. Figure 1 illustrates the two steps used to analyze the HCD activity outputs. In the first step, all outputs from each HCD activity were individually analyzed after each session to identify pregnancy and postpartum needs. In the second step, once all sessions were completed, the collected data were combined and analyzed to formulate key recommendations of integrating the maternal medical home and pathways community HUB.

**Figure 1:**
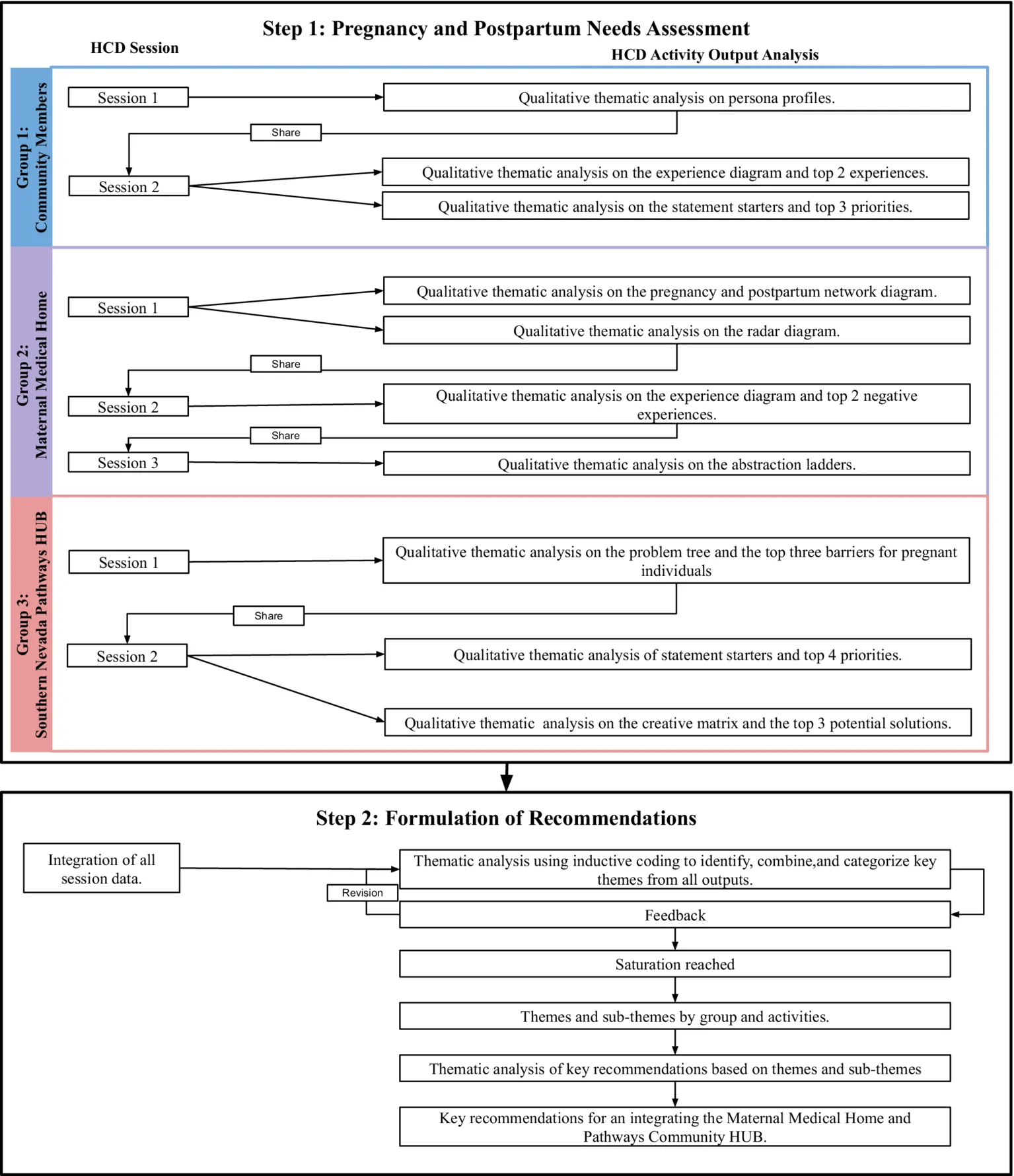
Flow chart of data analysis for human-centered design activities and integration activities.

#### 2.7.1 Step 1: pregnancy and postpartum needs assessment

A qualitative thematic analysis was conducted on the data collected from each group’s HCD session outputs prior to their subsequent sessions, as shown in Figure 1. Three authors (GB, ACS, AS) carried out the analysis to identify needs related to pregnancy and postpartum care, examining the frequency, complementarity, expansion, and divergence of themes across the outputs (25). For Group 1, the persona profiles that were collected in session 1 were thematically analyzed immediately post session and findings were presented at the beginning of session 2. The experience diagrams and statement starters collected from session 2 were thematically analyzed post session and results were shared with the group digitally. For Group 2, the pregnancy and postpartum network diagram and the radar diagram collected from session 1 was thematically analyzed post session and findings were presented at the beginning of session 2. The experience diagram collected in session 2 was thematically analyzed post session and results were shared at the beginning of session 3. The abstraction ladder collected in session three were thematically analyzed post session and shared with the group digitally. For Group 3, the problem tree collected from session 1 was thematically analyzed immediately post session and results were shared at the beginning of session 2. The statement starters collected from session 2 were thematically analyzed post session and shared with the group at the beginning of session 3. The creative matrix from session 3 was immediately thematically analyzed post session and shared with the group digitally. A description of session outputs and analysis methods for each group per session is outlined in Tables 1–3.

#### 2.7.2 Step 2: formulation of recommendations

After all sessions were completed for each group, all data collected from the outputs were integrated and analyzed to formulate key recommendations. Two authors (GB, AS) conducted a thematic analysis utilizing inductive coding to identify, combine, and categorize the key themes that emerged from all outputs (Figure 1). Coding happened in an iterative process, where analysis was conducted by one author (AS) and then results were reviewed by another author (GB) to provide feedback, then revised. This process continued until they reached saturation, which is when no new themes or codes could be identified (26). Once the final list of themes and sub-themes by group and activity were identified, they were further analyzed by five authors (GB, TC, NG, ACS, AS) to guide the formulation of key recommendations.

## 3 Results

A total of 35 individuals participated across the three HCD groups (Table 4). Group 1, the Community Member Group, included six participants, with equal representation of pregnant women/ new mothers (*n* = 3, 33%), community health workers (*n* = 3, 33%), and doulas (*n* = 3, 33%). Group 2, the Maternal Medical Home Group, included 18 participants, of whom half were physicians/providers (*n* = 9, 50%), followed by consultants (*n* = 6; 33%) and program evaluators (*n* = 3, 17%). Group 3, the Southern Nevada Pathways HUB Group, included 11 participants and was primarily composed of community organization representatives (*n* = 6, 54%), followed by SNVHUB care coordinators (*n* = 3, 27%), a birth support provider (*n* = 1, 9%), and a social service provider (*n* = 1, 9%).

**Table 4:** Characteristics of HCD participants, 2024.

Category	Frequency (n)	Total (%)
Group 1: community member group		
Pregnant and/or new mother	2	33.3
Community health worker	2	33.3
Doula	2	33.3
*Total*	6	100
Group 2: maternal medical home group		
Physician/Provider	9	50.0
Consultants	6	33.3
Program evaluator	3	16.7
*Total*	18	100
Group 3: Southern Nevada Pathways HUB (SNVHUB) group		
Birth support provider	1	9.1
Social service provider	1	9.1
SNVHUB care coordinator	3	27.3
Community Organization Representative	6	54.5
*Total*	11	100

This table includes the distinction between the three groups of participants and the individuals included in each group.

### 3.1 Pregnancy and postpartum needs assessment

A total of 10 outputs were generated from the 17 HCD activities conducted with each group to explore the needs related to pregnancy and postpartum care (Tables 1–3). Each output revealed key findings reflecting the specific needs during pregnancy and the postpartum period.

#### 3.1.1 Group 1 key findings

Group 1 engaged in a total of six activities within two HCD sessions, which resulted in three outputs (Table 1). The first output was individual persona profiles created by the participants. The findings were the themes of struggles and goals of pregnant individuals that were generated from the personal profiles. The themes that emerged the most from the persona profiles include goals for delivery, availability of resources, and experiencing misinterpretation. The second output was an experience diagram that mapped out the experiences of pregnant individuals with the healthcare system by trimester. The findings were themes of these experiences and the top two negative experiences that the participants wanted to move forward with, which were “no preparation for mom after birth” and “lack of resources.” The third output was a list of priority statements based off of the top two negative experiences from output two. The final finding was three priority statements that the community believed the model should consider including, “how might we create cultural competency/humility within hospitals and clinics,” “how might we educate mothers on how to take care of themselves after birth,” and “how might we educate mothers on their bodies.”

#### 3.1.2 Group 2 key findings

Group 2 engaged in a total of six activities within three HCD sessions, which resulted in four outputs (Table 2). The first output was a diagram of the individuals who are involved in a pregnant individual’s pregnancy journey. The findings from this output included nine different groups of stakeholders, including those in social support, build environments, social services, pregnancy specialists, other specialists, mental health, physicians, social needs, and clinical staff. The second output includes a radar diagram that shows the primary, secondary, and tertiary priorities that the participants had for pregnant patients. It was found that the most common primary concern was access to care, the most common secondary concerns were learning and education surrounding pregnancy and emotional support during and after delivery. The third output includes an experience diagram that mapped out the experiences of pregnant individuals with the healthcare system by trimester. The findings were the themes of pregnant individuals’ experiences and the top two experiences that the participants wanted to move forward with, which were “health education for patients and families” and “improving access to care and resources to decrease financial burden.” The last output were two abstraction ladders that included the “whys” and “hows” of the experiences they identified in the previous output. These abstraction ladders showed us what exactly the maternal medical home group wanted out of the model.

#### 3.1.3 Group 3 key findings

Group 3 engaged in a total of six activities within two HCD sessions, which resulted in three outputs (Table 3). The first output included a problem tree that depicted the root causes and impacts that contribute to barriers to care for pregnant individuals. The findings included themes of root causes and barriers and the top three barriers that the participants wanted to focus further on, including “limited resources,” “lack of support system,” and “no understanding what programs and services exist.” The second output was a list of priority statements based on two top barriers from output one. The final finding was themes of priority statements and the top four statements that the pathways integration group believed the model should consider including, “how might we engage the whole family in prenatal care,” “how might we create peer support within clinics,” “how might we teach more self-reliance, confidence, and self-efficacy,” and “how might we use community health workers to increase support.” The last output was a creative matrix that depicted potential solutions to two statements from output two. The final findings included themes of different solutions and the two solutions they wanted the model to focus on including “recruit and train folks from the community you are trying to serve” and “offer training, classes, and support in multiple ways in-person, virtually, one-on-one, and group.”

### 3.2 Formulation of recommendations

There were 11 key themes of needs that emerged from all HCD outputs, including (1) social support, (2) social determinants of health, (3) needs assessment, (4) access to resources, (5) medical, (6) pregnancy specialist, (7) education, (8) discrimination, bias, and cultural humility, (9) quality of care, (1) coordination of care, and (11) sustainability/advocacy (Table 5). These themes were then formulated into 15 key recommendations within four major categories for developing an integrated care coordination demonstration model (Table 6). The first major category was to consider pregnancy and postpartum needs within the integrated model and includes 6 key recommendations. Through the analysis of the key findings, it became clear that in order to develop a model that is fit for pregnant and postpartum women, the model must be centered around their needs. Key recommendations under this category include prioritizing what is necessary for pregnant and postpartum women to achieve optimal health outcomes for themselves and their infants. Examples of recommendations under this category include “we need to center the pregnant and postpartum women and focus on their social support” and “we should focus on supporting the well-being of mom and the baby by not only focusing on their clinical needs but also their social needs.” The second major category was to consider pregnancy and postpartum specialist needs within the model and includes 3 key recommendations. Through the key findings, it was identified that pregnant and postpartum women are in contact with a variety of specialists throughout their journey (e.g., midwives, doulas, genetic counselors, high-risk practitioners, lactation consultants, perinatal mental health therapists, and childbirth educators). Thus it is essential to consider some of their needs so that they can provide the best care to their patients. Some key recommendations under this category include “We need to ensure that there is a common language shared between all pregnancy and postpartum specialists” and “we need to ensure unbiased care by developing trusting relationships with providers, and safety as the top priorities.” The third major category is to consider the community system needs within the integrated model and includes 3 key recommendations. Key findings highlighted that many pregnant and postpartum women seek care or utilize resources outside of healthcare systems, including from social and community services. Therefore, we found that it was important to consider some of the needs of the community system, since this is another source of support for them. Some key recommendations from this category include “we need to create coordinated outreach systems with Community Based Organizations to reach the most vulnerable populations” and “we need to increase limited resources by creating culturally congruent care through diversifying the workforce and increasing availability and use of reimbursement and other funding models to support community embedded work.” Lastly, the fourth major category is to consider the healthcare system needs and includes 3 key recommendations. Pregnant and postpartum women usually have the most healthcare interactions with the healthcare system, and thus in order to have a holistic approach to improving their care, the needs of the healthcare system must be considered. Some of the key recommendations from this category include “we need to provide training for implicit and explicit bias, as well as cultural competency and humility for clinical staff” and “we need to optimize the electronic health record to capture social determinants of health data and work with claims to reimburse physicians for social determinants of health screening.”

**Table 5:** Key themes and sub-themes from all human-centered design outputs, 2024.

Theme	Theme definition	Sub themes	Group 1			Group 2				Group 3		
			Personas	Experience diagramming	Statement starters	VIP map	Radar	Experience diagram	Abstraction ladder	Problem tree	Statement starters	Creative matrix
Social Support Needs	The network of individuals that provide either instrumental support, emotional support, or informational support (e.g., family, partners, communities) to pregnant and postpartum women (43).	Family				x		x			x	
		Community			x	x	x	x			x	
		Partner	x									
Social Determinants of Health Needs	Non-clinical conditions (e.g., transportation, food insecurity, housing, finances) that can directly or indirectly impact the health of pregnant and postpartum women (7, 8, 40, 41).	Public Services and Infrastructure				x						
		Transportation			x					x		
		Food Insecurity					x	x		x		
		Housing					x	x		x		
		Substance Use								x		
		Finances		x			x	x	x	x		
Health Needs Assessment Needs	Provision of assessing or screening the different health needs of pregnant and postpartum women (e.g., social needs, mental health needs, health care needs) to be able to properly care for them (44).	Social Needs Assessment				x			x		x	
		Mental Health Needs Assessment			x	x	x				x	
		Health Care Needs Assessment								x		
Access to Resources Needs	Pregnant and postpartum women’s ability to obtain and utilize the necessary services, support, education, or information to assist with their needs (45).	Providing Resources	x		x							
		Availability of Resources	x	x	x						x	
		Social Services Resources				x	x					
		Provide Resources Virtually and In-Person										x
Medical Needs	Healthcare services, supplies, or staff needed to assist pregnant and postpartum women in diagnosing, treating, or preventing adverse health outcomes (46).	Physicians				x						
		Clinic Staff				x						
		Creating Peer Support with Clinics									x	
		Labs and Scans					x	x				
Pregnancy Specialist Needs	The different types of specialists that pregnant and postpartum women are in contact with throughout their journey, including but not limited to physicians, midwives, nurses, doulas, social workers, psychologists, lactation consultants, and psychiatrists (42).	Doulas				x					x	
		Midwife				x					x	
		Lactation Consultant				x					x	
		High-Risk Pregnancy Specialists				x					x	
Education Needs	Education that allows the transfer of knowledge, information, and skills that will enable pregnant and postpartum women to manage their own health, make informed decisions, and improve their well-being (47).	Breastfeeding Education		x	x						x	
		Increasing Patient Self-Efficacy									x	
		High Literacy and Healthcare Resources		x	x		x		x	x	x	
		Pregnancy Education		x				x				x
		Mental Health and Psychological Medication Education					x					x
		Patient Education on Bodies and Decision-Making			x							
		Education for Partners										x
Discrimination and Bias Needs	Discrimination and bias can often lead to pregnant and postpartum women receiving unequal treatment in care, which could lead to health inequities (48).	Provider Training on Bias			x							
		Discrimination/Bias	x	x			x			x		
		Cultural Competency/Humility of Care Training for Providers			x		x			x		
Quality of Care Needs	The appropriateness and effectiveness of the care that pregnant and postpartum women receive during their pregnancy (49).	Early Engagement in Prenatal Care		x			x	x				
		Reduce Disparities in Pregnancy outcomes							x			
		Patient Goals for Delivery	x	x				x				
		Patient Goals Related to Child/Family	x					x				
		Birth Complications and Preterm Birth	x									
		Appointments/Care Accessibility		x	x			x				
		Postpartum Care		x	x							
		Patient Empowerment/Positive Patient Experience	x	x	x		x			x		
Coordination of Care Needs	The organization of pregnant and postpartum women’s care across numerous pregnancy specialists and providers.	Provider Training on Mental Health Referrals			x							
		Continuity of Care	x				x					
		Collaboration Among Social Service Providers					x		x		x	
Sustainability/ Advocacy Needs	Ensuring that all strategies to improve pregnancy and postnatal health outcomes are able to be maintained, which could be achieved through advocacy.	Policy									x	
		Data and Reporting							x			

This table shows the 11 different key themes of needs that emerged from all human-centered design outputs, the sub themes that fall within each theme, and which activity and group they came from.

**Table 6:** Key recommendations for developing an integrated maternal health services model, 2024.

Category	Key recommendation	Representative quote(s)
Consider these pregnancy and postpartum needs within the Model	We need to center the pregnant and postpartum women and focus on their social support (i.e., partners, families, communities/neighborhoods, employers, etc.).	“Positive patient experience (i.e., patient has a say in how things go with their pregnancy and delivery, open communication with patient” -Group 2 “Patient autonomy. Allowing each patient ot voice what they need, meeting individual needs” -Group 2 VIP mapping identified important networks of individuals within the pregnancy journey (e.g., partners, families, father of the baby, community/neighbors, employers, teachers, spirituality/faith. -Group 2
	We should focus on supporting the well-being of mom and baby by not only focusing on their clinical needs but also their social needs.	“How might we connect mothers to mental health care” -Group 1 “Social/emotional support (during/after)” -Group 2 “Immediate household needs (bills, food, kids, childcare, school)” -Group 2
	We need to have data to identify social determinants of health gaps and address disparities through health education and access to services to address health related social needs for families and patients.	“How might we help patients be more health literate” -Group 1 “How might we identify health problems and social determinants of health early and ongoing” -Group 2 “How might we increase health literacy” -Group 2
	We should consider educating and empowering parents and caregivers to utilize “BRAIN” (Benefits, Risks, Alternatives, Intuition, Need more time/Nothing).	“How might we educate mothers on ‘BRAIN’” -Group 1 “Patient autonomy. Allowing each patient ot voice what they need, meeting individual needs” -Group 2
	We need to increase support systems by offering training, classes, resources, and support for pregnant individuals, new moms, and their families in multiple ways (i.e., in-person, virtual, one-on-one).	“How might we provide education on researcher to a mother after birth”-Group 1 “How to educate mothers on the importance of postpartum care” -Group 1 “How might we educate mothers on what to look for after birth, and when they should seek intention” -Group 1 “Offer free training.classes, support in multiple ways in person/virtual, one on one/ group” Group 3 “Childbirth, breast/chest feeding education” -Group 3 “Create policies that require staff to provide information and resources to pregnant people and their families” -Group 3
	We need to create a system to follow-up with moms after birth to check in on their perinatal and social–emotional health.	“How might we provide education and resources to a mother after birth” -Group 1 Follow up after birth and 3-months/ 6-months to see how things are going” -Group 3
Consider these pregnancy and postpartum specialists’ needs within the model.	We should consider all pregnancy and postpartum specialists in the care team (i.e., midwives, doulas, genetic counselors, high-risk practitioners, lactation consultants, perinatal mental health therapists, and childbirth educators).	“How might we increase transportation to specialty care and regular appointments” - Group 1 VIP mapping identified many specialist during the pregnancy journey including midwives, doulas, genetic counselors, high risk practitioners, lactation consultants, behavioral health specialists, psychologist, physical therapists, among others -Group 2
	We need to ensure that there is a common language shared between all pregnancy and postpartum specialists.	“Reducing the same questions that are asked by different shift nurses/physicians (communication between staff” -Group 1
	We need to ensure unbiased care by developing trusting relationships with providers, and safety as the top priorities.	“In what ways can we train providers to provide a welcoming environment” -Group 1 “Trusting relationship with provider” -Group 2
Consider these Community Systems Needs within the Model	We need to consider the social (i.e., social workers, care managers, care coordinators, WIC/SNAP, legal agencies) and community (i.e., schools, childcare, housing, delivery environment, transportation, parks, YMCAs) services available within a pregnant and postpartum individuals community and how to communicate these services with pregnant individuals, new moms, and their families (i.e., resource sheet).	“How might we utilize groups that are already available in the community” -Group 1 VIP mapping identified many social and community services important in a pregnancy journey including schools, childcare, care coordinators, social workers, case managers, agencies, among others- Group 2
	We need to create coordinated outreach systems with Community Based Organizations to reach the most vulnerable populations (i.e., lack of insurance).	“How might we utilize groups that are already available in the community” -Group 1 “How might we collaborate with community based organizations (profit and non-profit partners)” -Group 2 “Use the Southern Nevada Pathways Community HUB” -Group 3
	We need to increase limited resources by creating culturally congruent care through diversifying the workforce (i.e., community health workers, recruiting individuals from the community) and increasing availability of and use of reimbursement and other funding models to support community embedded workforces (e.g., CHWs, doulas, etc.).	How might we meet the needs of those who are unable to find an agency that they feel comfortable going to/ look like them” -Group 3 “Diverse community health workers” -Group 3 “Recruit and train folks from the community you are trying to serve” -Group 3 “Ensure there is enough funding to sustain start up efforts and operating costs for the future” -Group 3
Consider these Healthcare Systems Needs within the Model	We need to provide training for implicit and explicit bias, as well as cultural competency and humility for clinical staff.	“How to create cultural competency/ humility within hospitals/clinics” -Group 1 “In what ways can we train providers on implicit and explicit bias” -Group 1 “Cultural sensitivity/ awareness, understanding your patients and how to help them based on their needs” -Group 2 “Unbiased care” -Group 2
	We should consider strategies to increase access to care and patient engagement during the first trimester of the pregnancy (i.e., decrease financial burdens, identify social determinants of health early and ongoing, improve, and promote healthy pregnancy, and establish partnerships with community based organizations).	“How might we make access to care available to the most vulnerable populations”- Group 2 “How might we provide access to consistent clinical care” -Group 2 “How might we ensure that everyone has a reliable and consistent pay source” -Group 2
	We need to optimize the electronic health record to capture social determinants of health data and work with claims to reimburse physicians for social determinants of health screening.	“How do we utilize data to understand health and socioeconomic disparities” -Group 2 “How might we optimize electronic health record data”-Group 2 “How might we perform risk assessments for underserved populations” -Group 2

This table includes the four categories of key recommendations and the recommendations that are within them.

## 4 Discussion

This study utilized HCD to engage diverse voices in documenting the needs of pregnant women and mothers in Clark County, Nevada. This approach also highlighted barriers that clinical and social providers face in providing services to pregnant women and recent mothers. Through the HCD activities, key themes of pregnancy and postpartum needs, and service barriers were identified, including social support needs, social determinants of health needs, conducting need assessments, access to resources, medical needs, pregnancy provider’s needs, educational needs, discrimination and bias training needs, quality of care needs, coordination of care needs, and sustainability or advocacy needs. These key themes were then used to develop recommendations for designing a model. The HCD was a feasible and relatively low-burden community-engaged approach to place the community at the center of designing a model.

The recommendations that resulted from the key themes corroborate with prior literature as ways to promote a healthy pregnancy and enhance pregnant women and mothers’ health outcomes, which ultimately improves their infants’ health outcomes (27–38). Our study innovates by organizing these key themes into actionable recommendations to inform the design of a model. These recommendations fall within four major categories.

First, the model must center the pregnant women and mothers and consider their needs. Therefore, it is recommended that the model consider developing strategies to increase or enhance maternal support systems. A critical component to promoting healthy maternal outcomes is women’s support systems, including healthcare, access to resources, and social support systems (32, 36). Studies have found that inadequate social support during pregnancy is associated with increased risk for depression and anxiety, and adverse health outcomes for both the mother and infant (32). However, strong social support enhances the pregnancy and motherhood experience by building relationships with different health professionals, gaining new knowledge, and developing meaningful peer connections (32). Another critical component to help pregnant women and mothers achieve optimal health outcomes is addressing not only their clinical needs but also their social needs (30). Social needs, or social determinants of health, are nonclinical conditions that can disrupt daily life and well-being (30, 39). This can include food or housing insecurity, lack of transportation, systemic racism, unequal access to healthcare, and disparities in care quality, which influence health outcomes (8, 40, 41). Although social determinants of health plays a substantial role in the health and well-being of pregnant women and their infants, screening and addressing social determinants of health needs are not a standard of care for most, if not all, prenatal care (30). Addressing social needs includes identifying needs and providing access to critical resources and support during and after pregnancy (32, 33, 36). Therefore, it is recommended that the model consider focusing on not only addressing clinical needs, but also the social needs of pregnant women and mothers. Lastly, an essential need for the best maternal outcomes is postpartum care (31). The postpartum period is a critical window for care, as women and their infants are at higher risk for adverse outcomes and complications (31). There is a need for care and support during this period to ensure women and their infants reach their optimal health outcomes (31). Thus, it is recommended that the model consider developing a follow-up system to check on the perinatal and social–emotional health of women (31).

Second, the model should consider the needs of various pregnancy providers. Pregnant women and mothers are in contact with many different providers during and after their pregnancy (42). These providers include but are not limited to physicians, midwives, nurses, doulas, social workers, psychologists, lactation consultants, and psychiatrists (42). As pregnant women and mothers are communicating and connecting with numerous providers during their journey, it is imperative that there is a common language between all maternal providers to eliminate any confusion (27). A lack of common knowledge can lead to poor communication, which can lead to negative care experiences, confusion, and inefficient use of providers’ time (27). Therefore, it is recommended that the model consider addressing this language gap barrier between maternal providers. It is also essential that a trusting relationship is built between these maternal providers and pregnant women (28, 29). Strong patient-provider relationships foster optimal treatment outcomes, greater satisfaction, patient participation in their health, adherence to recommendations, sense of support and reassurance, and better self-care, confidence, and emotional state (28, 29). Thus, the model should consider developing strategies to help maternal providers improve their skills in building relationships with pregnant women.

Third, the model should consider the needs of the different established community systems. Most pregnant women and mothers acquire prenatal care from providers in healthcare systems (42, 51). However, others seek care from social and community services, especially if that is what is affordable and accessible to them (37, 42, 51). Pregnant women and mothers may have some community-based organizations available to them to assist with different pregnancy needs (37). Other community services available could include community-based doulas, midwives, birth centers, group prenatal care, community health workers, and pregnancy medical homes (37). These services are developed for those who wish to experience cost-saving, unbiased care that they otherwise might not get within the healthcare system (37). Therefore, it is recommended that the model consider how to communicate these services to pregnant women and mothers and how to work with the community-based organizations to reach the most vulnerable populations (37). Additionally, to make these community services more accessible to a broader maternal population, it is recommended that the model consider strategies to expand or improve reimbursement or funding models (37). Since part of these community services is to provide unbiased care, it is also recommended that the model consider strategies to diversify the workforce. Studies have found that when pregnant women and mothers are treated by providers who look, speak, or relate to them, it could result in improved care experiences, access to care, quality of care, and better health outcomes (38).

Lastly, the model should consider several healthcare system needs. Implicit and explicit bias within healthcare systems is a contributing factor to poor maternal and infant health outcomes and racial disparities in the United States (34). Implicit bias is when a provider unconsciously stereotypes and has attitudes towards specific populations, which influences their care, decisions, and actions towards patients (34, 35). Alternatively, explicit bias is similar, but these providers are aware and conscious of it (35). Therefore, it is recommended that the model consider providing implicit and explicit bias training to the providers that they plan to include in their model. As mentioned previously, social determinants of health plays a substantial role in the health and well-being of pregnant women and their infants; however, screening and addressing social determinants of health needs are not a standard of care for most, if not all, prenatal care (30). The American College of Obstetricians and Gynecologists recommends a comprehensive risk assessment during prenatal and postpartum care that should assess both clinical and nonclinical factors (e.g., social determinants of health) to reduce pregnancy-related deaths and ultimately maternal mortality (53). Thus, it is recommended that the model consider optimizing the electronic health record to capture social determinants of health data and reimburse providers for their screenings.

The HCD activities that were conducted resulted in a unique set of recommendations to design a model to improve the experiences of pregnant women and mothers in Clark County, Nevada. There is a limited body of evidence on how to meaningfully engage the community and other interest-holders within the context of maternal and child health, and this study contributes to that (19, 43). The benefits of utilizing HCD included the ability to gather input from a diverse set of voices, including physicians, community-service providers, pregnant women, and mothers with different perspectives on the experiences of pregnant women and mothers. These activities focus on centering pregnant women and mothers, engaging with them, and listening to their needs and those of the professionals working with them. Doing this contributed to the appreciation and ownership of the design and development of a new model. Likely, it highlighted the direction in which the model should be designed. However, facilitating these activities did come with some challenges. First, as there were three different groups, it was difficult to coordinate schedules between participants to conduct one to two-hour activities. Due to this, facilitators decided to offer in-person and virtual meeting options to participants, but those came with challenges of their own. For in-person meetings, transportation and weather issues prevented some individuals from attending the activities. Particularly for the community members and the Maternal Medical Home groups, facilitators tried to alleviate these issues by conducting activities in spaces and areas that were easily accessible to them or by offering virtual options. For the activities that resulted in virtual meetings, facilitators met with technical challenges with Mural and a lack of engagement. Facilitators tried to improvise when Mural would not let participants share their ideas on its platform, instead having them openly discuss their ideas with the group. Another limitation of this co-creation process was that participants were divided into three groups, with each group engaging in different activities tailored to their roles and experiences. This may have limited opportunities for direct dialogue and collective discussions across groups. However, this approach was intentionally designed to help mitigate power differentials that can arise when individuals with varying levels of expertise and experience engage in the same setting. Conducting activities separately may have created a more comfortable environment for participants to share their perspectives openly and honestly. To ensure that the findings reflected the breadth of perspectives represented across all groups, the research team took all the data collected from each activity and integrated them during the analysis process. Recommendations developed were based on the integrated findings rather than the views of any single group. We believe this process strengthened the comprehensiveness of the recommendations while maintaining a participatory approach that was attentive to the issues of power and representation. Lastly, since there were so many activities and groups, the researchers had to put in considerable effort to analyze everything that was collected.

Some limitations should be taken into consideration when interpreting and generalizing the results from this study. First, since this was technically a cross-sectional study, findings only represent the knowledge of experiences of the group members at that moment in time. This may have resulted in not adequately capturing the most significant experiences of pregnant individuals and mothers, and may not have resulted in a recommendation for these specific experiences. Second, as this was qualitative research, the findings are contextual and may have been influenced by the researchers’ experiences and perspectives during data interpretation. However, the research team strived to reduce this influence by engaging multiple research team members in the analysis and review of findings, and findings were presented back to participants for review. Lastly, although a diverse set of voices was engaged in these activities, the research team may have missed relevant individuals with maternal care experiences; however, we are still provided with insights that resulted in recommendations that are still meant to help this population. Despite these limitations, this study offers meaningful insights into maternal needs that informed the development of recommendations aimed at supporting this population. Notably, this work represents an innovative effort to apply evidence-based methods in engaging diverse voices to inform the design and development of a model.

Moving forward, all participants from each group of the HCD activities have continued to assist in the design and development of the model. The recommendations from this study will be used to make decisions on the priority needs, model components, and the direction of the model. Once developed, the model will transition to implementation within an academic obstetric clinic and a Pathways Community HUB, with clinic and HUB staff responsible for integrating the model into their respective workflows and service delivery processes. Continued collaboration among the research team, clinic staff, HUB staff, and community partners will support the transition from the recommendations generated through the HCD activities to implementation in practice. The use of the recommendations to design, develop, and implement this model is expected to result in more comprehensive care and meet the needs of pregnant women and mothers, which can lead to optimal health outcomes for themselves and their infants. Additionally, implementation of the model may identify opportunities for broader systems and policy changes, such as expanding nonclinical screening practices in obstetric settings and advocating for reimbursement mechanisms to support these screenings.

## 5 Conclusion

This study describes an innovative approach to engage a diverse set of voices to illuminate the needs of the maternal population and generate recommendations to develop a model to address these needs. To develop a model that aims to improve maternal outcomes, it is imperative that the model consider the needs of pregnant women and mothers, maternal providers, community systems, and healthcare systems. This includes enhancing maternal support systems, screening and addressing clinical and nonclinical needs, ensuring that a common language is shared between maternal providers, building provider-patient relationships, working with community-based organizations and services, and providing implicit and explicit bias training for maternal providers. We recommend that future studies use these recommendations in the design and development of maternal health service models. Additionally, future studies could use HCD activities to illuminate the needs of their target populations to help develop recommendations or strategies to improve maternal-child health outcomes.

## Data Availability

The raw data supporting the conclusions of this article will be made available by the authors, without undue reservation.
